# Heat generation/absorption effect on natural convective heat transfer in a wavy triangular cavity filled with nanofluid

**DOI:** 10.1038/s41598-023-48704-2

**Published:** 2023-12-01

**Authors:** Tarikul Islam, Md. Nur Alam, Shafiullah Niazai, Ilyas Khan, Md. Fayz-Al-Asad, Sultan Alqahtani

**Affiliations:** 1 CMUP, University of Porto, Porto, Portugal; 2 Department of Mathematics, Bangabandhu Sheikh Mujibur Rahman Science and Technology University, Gopalganj, 8100 Bangladesh; 3 Department of Mathematics, Pabna University of Science and Technology, Pabna-6600, Bangladesh; 4 Department of Mathematics, Education Faculty, Laghman University, Mehtarlam City, Laghman 2701 Afghanistan; 5 Department of Mathematics, College of Education, Majmmah University, 11952 Al-Majmaah, Saudi Arabia; 6 Department of Mathematics, American International University-Bangladesh, Kratoli, Khilkhet, Dhaka-1229, Bangladesh; 7 Department of Mathematics, Bangladesh University of science and technology (BUET), Dhaka-1000, Bangladesh; 8 College of Engineering Mechanical Engineering Department, King Khalid University, Abha, Saudi Arabia

**Keywords:** Engineering, Mathematics and computing, Nanoscience and technology

## Abstract

This study is numerically executed to investigate the influence of heat generation or absorption on free convective flow and temperature transport within a wavy triangular enclosure filled by the nanofluid taking the Brownian effect of nanoparticles. The water (H_2_O) is employed as base fluid and copper (Cu) as nanoparticles for making effective Cu–H_2_O nanofluids. The perpendicular sinusoidally wavy wall is cooled at low temperature while the horizontal bottom sidewall is heated non-uniformly (sinusoidal). The inclined wall of the enclosure is insulated. The governing dimensionless non-linear PDEs are executed numerically with the help of the Galerkin weighted residual type finite element technique. The numerically simulated results are displayed through average Nusselt number, isothermal contours, and streamlines for the various model parameters such as Hartmann number, Rayleigh number, heat generation or absorption parameter, nanoparticles volume fraction, and undulation parameter. The outcomes illustrate that the temperature transport rate augments significantly for the enhancement of Rayleigh number as well as nanoparticles volume fraction whereas reduces for the increment of Hartman number. The heat transfer is significantly influenced by the size, shape, and Brownian motion of the nanoparticles. The rate of heat transport increases by 20.43% considering the Brownian effect for 1% nanoparticle volume. The thermal performance increases by 8.66% for the blade shape instead of the spherical shape of nanoparticles. In addition, heat transfer is impacted by the small size of nanoparticles. The thermal transport rate increases by 35.87% when the size of the nanoparticles reduces from 100 to 10 nm. Moreover, the rate of heat transmission increases efficiently as the undulation parameter rises. It is also seen that a crucial factor in the flow of nanofluids and heat transmission is the heat generation/absorption parameter that influences temperature distribution, heat transfer rates, and overall thermal performance.

## Introduction

The investigation of natural convection using nanofluids remains a promising field for raising heat transfer efficiency and thermal management in a variety of applications. Natural convective heat transfer within a triangular cavity has widespread applications in numerous industrial, and engineering systems like heat exchangers, ventilation systems, solar collectors, fire prevention, refrigeration, geothermal reservoirs, and so on. A number of researchers^1–7^ investigated the nanofluid under different circumstances. Aydin et al.^8^ investigated free convective flow into a rectangular enclosure warmed by one side and consoled from the roof. Holtzman et al.^9^ performed free convective laminar flow into an isosceles triangle cavity warmed from the bottom side. Buongiorno^10^ investigated convective transport in nanofluids. Das et al.^11^ observed about nanofluids and their applications for science and technology. Free convective within a triangle-shaped cavity by an extending isothermal cooker was observed by Varol et al.^12^. Basak et al.^13^ investigated free convection simulation for both uniform and non-uniform systems within an isosceles triangle enclosure. Kent^14^ observed a free convective numerical solution in a triangle chamber where the base sidewall is cooled and the inclined wall is heated. Kamiyo et al.^15^ performed an extensive study on natural convective flow within a triangular cavity. Bhuvaneswari et al.^16^ performed hydromagnetic flow within a square-shaped cavity using a non-uniform heated sidewall.

The natural convection in a square chamber filled with a water-based nanofluid (water containing Cu particles) was explored by Tayebi et al.^17^. Tayebi and Ali J. Chamkha^18^ researched to comprehend the properties of natural convection heat transfer and flow using Cu/H_2_O in the square domain subjected to a horizontal magnetic field and different conductive boundary configurations. The natural convection flow mechanism and heat exchange under a magnetic field within a concentric circular annulus between a heat-generating conductive internal cylinder and an isothermally cold exterior cylinder filled with a CNTs-water-based nano-liquid were examined by Tayebi et al.^19^. The study conducted by Alsabery et al.^20^ examined the creation of entropy and mixed convection flow within a wavy-walled enclosure that contains a heat source and a rotating solid cylinder. The influence of natural convection within a square cavity, in addition to the presence of a conducting solid block and a corner heater, was numerically examined by Alsabery et al.^21^. The heat and flow exchange properties in a new configuration saturated with a Ag–MgO hybrid nanofluid were explored numerically by Hussain et al.^22^. Nasrin^23^ investigated MHD combined convective flow into the wavy cavity by account of Joule heating. Bhardwaj et al.^24^ performed an exploration of natural convective temperature disposal with entropy generation into a triangle-shaped cavity. Hamida and Charrada^25^ studied temperature generation or absorption effects upon free convective temperature flow into a square cavity using nanofluids including the magneto-hydrodynamic effect. Suvash et al.^26^ investigated free convective flow within a triangle-shaped cavity using uniform thermal boundary conditions.

Several common types of nanoparticles are obtainable for commercial purposes like Al_2_O_3_, Fe_3_O_4_, Cu, TiO_2_, and so on. Some of these nanoparticles are also effectively employed to enhance thermal performance. Hydromagnetic natural convective temperature flow within an isosceles triangle shape cavity using nanofluid was investigated by Rahman et al.^27^. Mirabedin^28^ performed computational simulations on natural convective flow into a right-angled triangle-shaped cavity. Alam et al.^29^ studied numerically convective temperature flowing of micropolar fluid including heat generation/absorption. Triveni et al.^30^ studied free convective flowing into the triangle cavity using the different locations of the cold wall. Boulahia et al.^31^ performed combined convection temperature disposal within a square enclosure charged with nanofluid using triangle-shaped warmed-up obstacles. Uddin et al.^32^ studied computational simulation numerically for convective heat flow into nanofluid. Unsteady convective MHD numerical impact of sinusoidal warmed boundary condition with Fe_3_O_4_-water ferrofluid was performed by Alam et al.^33^. Al-Asad et al.^34^ studied hydrodynamics impacts of convective flow using a perpendicular fin within a wavy square cavity. Zahan et al.^35^ studied the combined magneto-hydrodynamic convective flow in a wavy cavity considering Joule heating. The magnetohydrodynamics natural convection and entropy generation of a Cu–water nanofluid constrained in a porous annulus was numerically discovered by Mourad et al.^36^. In order to examine the heat transport processes of a non-Newtonian hybrid nanofluid in an annular enclosure, Abderrahmane et al.^37^ explored the MHD convective transport. Alshare et al.^38^ investigated convection in a nanofluid-filled, lid-driven hollow exposed to a magnetic field.

An emerging magnetic field is also employed to control the mechanism in material manufacturing processes. Izadi et al.^39^ examined the thermal and flow characteristics by considering both uniform and nonuniform magnetic field effects as well as time-dependent heat sources. Izadi et al.^40^ also investigated the convective flow of Al_2_O_3_/water nanofluid an annulus. They reported the heat transfer coefficient is directly influenced by the Richardson number. Convection heat transfer rate improved when the single nano-fluid (water/aluminum oxide) inside the chamber's nano-particle volume fraction increased^41^. The behavior of phase change material (PCM) was also marginally affected by the volume fraction^42^. As the concentration of nanoparticles increases, the bottom heated wall's mean Nusselt number falls^43^. Izadi et al.^44^ also showed how the placement of the heat sources affects how quickly the melting front advances. The increase in the average Nusselt number and energy transport intensity is seen for the Rayleigh number^45^. Additionally, it has been noted that when the flow motion gets stronger due to convection, there is an increase in instability in the temperature and flow fields^46^. When the Rayleigh number rises, the rate of heat transfer increases^47^. The average Nusselt number rises to 61% for Ra = 10^5^ and 12% for Ra = 10^3^^48^. When the magnetic field is ignored (Ha = 0), the dimensionless melting time increases by 266% when the Ha is imposed up to 500^49^. In comparison to α = 60°, the average Nusselt number for the position of the heat source and sink (α =  − 60°) is up to 124.5% greater. The heat transfer coefficient rises by 11.45% at Re 4000 by adding 1% nanoparticle volume to the base fluid^49^. Tarikul et al.^50–56^ recently studied numerically MHD influence on free convective temperature distributions into the different nanofluids with different thermal conditions.

It appears from this study that there is a lot of scope for research on nanofluids under different thermal conditions for checking thermal performance which has specific applications such as solar collectors, cooling/heating systems, and so on. The main intention of the current research is to explore convective flow and thermal transport into a wavy triangle-shaped cavity utilizing the nanofluids with the presence of heat generation/absorption and sinusoidal thermal boundary conditions. The influence of various physical parameters such as undulation parameter, heat generation/absorption, Rayleigh number, nanoparticle volume, Hartmann number, nanoparticles shape and size, and Brownian motion and other flow behaviors of nanofluids on the fluid flow and heat transfer performance are numerically studied and discussed from the physical standpoint.

## Problem formulation

### Physical model

**Figure 1:**
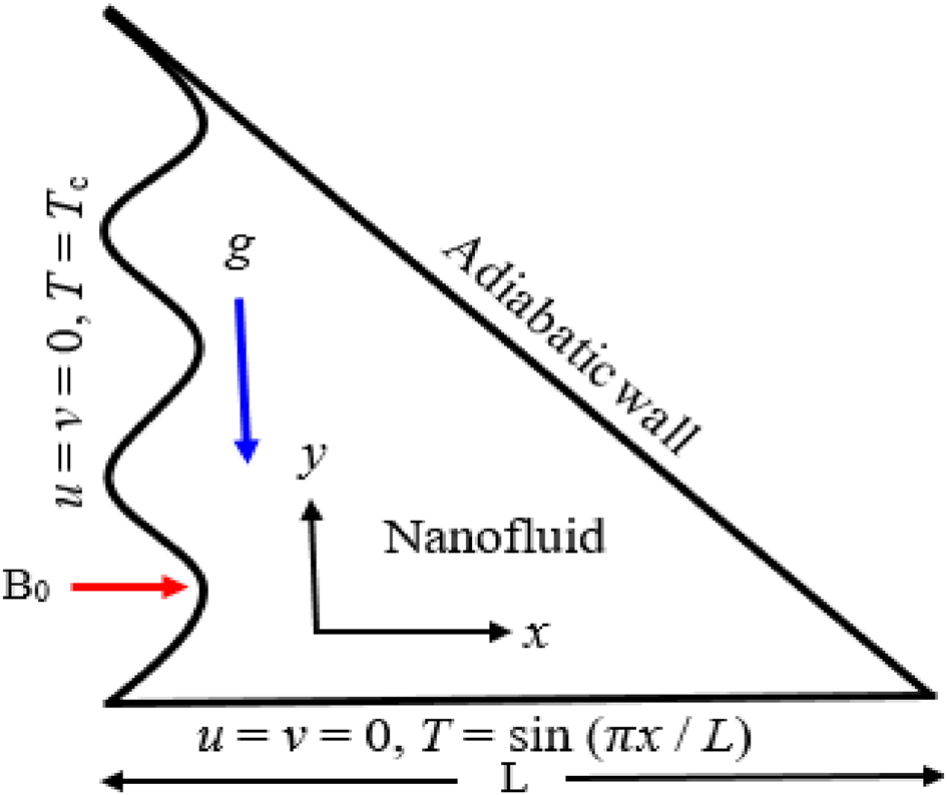
Schematic view for sinusoidal triangular enclosure.

A schematic view of the wavy triangular enclosure considered for the present research, boundary conditions, and the coordinates are displayed in Fig. 1. In this enclosure, the vertical sinusoidal wall is nursed at a low-temperature *T* = *T*_*c*_, whereas its horizontal wall is warmed sinusoidally $T = \sin \,(\pi x/L)$. The hypotenuse of the enclosure is insulated. The copper–water nanofluid (Cu–H_2_O) is employed in the enclosure where water (H_2_O) is taken as base fluid and copper (Cu) as nanoparticles. The strength (B_0_) of the uniform magnetic field is employed towards the horizontal direction and normal to the vertical wavy wall. *g* = (0, *g*) is the gravitational acceleration that works downward direction towards the *y*-axis. The nanofluid is considered to be laminar, viscous, and incompressible. The two-dimensional time-independent flow is also assumed. The horizontal wall measures the *x*-axis and the vertical wall measures the *y*-axis. Every rigid boundary is accepted like a no-slip wall. The characteristics of base fluid and nanoparticles have been given in Table 1. In the absence of the electric field (${\varvec{E}}$ = **0**), the interaction of the fluid flow with the magnetic field applied to it gives rise to the Lorentz force and the uniform magnetic force with ${\varvec{B}}=( {B}_{0}, 0, 0)$ can be written as follows:

**Table 1:** Thermophysical properties of the base fluid and solid particles (see^57,58^).

Base fluid/nanoparticles	*c*_p_ [Jkg^−1^K^−1^]	*ρ* [kgm^−3^]	*k* [Wm^−1^K^−1^]	*μ* [kgm^−1^s^−1^]	*β* × 10^−5^ [K^−1^]	*σ* [Sm^−1^]	Pr
H_2_O	4179	997.1	0.613	0.001003	21	5.5 × 10^−6^	6.8377
Cu	385	8933	400	–	1.67	5.96 × 10^7^	–

1 $${\sigma }_{nf}({\varvec{u}}\times {\varvec{B}})\times {\varvec{B}}={{\varvec{F}}}_{m}=(0, -{{\sigma }_{nf}B}_{0}^{2}v, 0)$$

The feature of the vertical wavy wall is employed to mimic the following model:

2 $$ X = A\,(1 - \cos (2\pi \lambda x)) $$

where *A* represents wave amplitude and *λ* represents undulation number.

### Mathematical model

The governing non-linear PDEs of conservation of mass, momentum, and energy for nanofluid for the aforementioned assumptions are displayed in the Cartesian coordinate system in dimensional form^57,58^:

3 $$ \frac{\partial u}{{\partial x}} + \frac{\partial v}{{\partial y}} = 0 $$

4 $$ u\frac{\partial u}{{\partial x}} + v\frac{\partial u}{{\partial y}} = - \frac{1}{{\rho_{nf} }}\frac{\partial p}{{\partial x}} + \frac{{\mu_{nf} }}{{\rho_{nf} }}\left( {\frac{{\partial^{2} u}}{{\partial x^{2} }} + \frac{{\partial^{2} u}}{{\partial y^{2} }}} \right) $$

5 $$ \rho_{nf} \left( {u\frac{\partial v}{{\partial x}} + v\frac{\partial v}{{\partial y}}} \right) = - \frac{\partial p}{{\partial y}} + \mu_{nf} \left( {\frac{{\partial^{2} v}}{{\partial x^{2} }} + \frac{{\partial^{2} v}}{{\partial y^{2} }}} \right) + \left( {\rho \beta } \right)_{nf} g(T - T_{c} ) - \sigma B_{0}^{2} \nu $$

6 $$ u\frac{\partial T}{{\partial x}} + v\frac{\partial T}{{\partial y}} = \alpha_{nf} \left( {\frac{{\partial^{2} T}}{{\partial x^{2} }} + \frac{{\partial^{2} T}}{{\partial y^{2} }}} \right) + \frac{q}{{(\rho c_{p} )_{nf} }}\left( {T - T_{c} } \right) $$

### Boundary conditions (dimensional)

7 $$ {\text{At the horizontal sidewall}}:\quad u = 0,\,\,v = 0,\,\,T = \sin \,(\pi x/L) $$

8 $$ {\text{At the vertical wavy sidewall}}:\quad u = 0,\,\,v = 0,\,\,T = T_{c} $$

9 $$ {\text{At the inclined wall}}:\quad u = 0,\,\,v = 0,\,\,\frac{\partial T}{{\partial x}} = 0 $$

### Thermophysical properties of nanofluids

The physical properties of nanofluids such as thermal diffusivity, specific heat, thermal expansion coefficient, viscosity, density, and electrical conductivity of nanofluids that appear in governing Eqs. (3)–(6) are given by the following formulations^57,58^:

10 $$ \alpha_{nf} = \frac{{k_{nf} }}{{\left( {\rho c_{p} } \right)_{nf} }} $$

11 $$ \left( {\rho c_{p} } \right)_{nf} = \left( {1 - \phi } \right)\left( {\rho c_{p} } \right)_{bf} + \phi \left( {\rho c_{p} } \right)_{sp} $$

12 $$ \left( {\rho \beta } \right)_{nf} = \left( {1 - \phi } \right)\left( {\rho \beta } \right)_{bf} + \phi \left( {\rho \beta } \right)_{sp} $$

13 $$ \mu_{nf} = \mu_{bf} \left( {1 - \phi } \right)^{ - 2.5} $$

14 $$ \rho_{nf} = \left( {1 - \phi } \right)\rho_{bf} + \phi \rho_{sp} $$

15 $$ \sigma_{nf} = \frac{{\sigma_{sp} + 2\sigma_{bf} - 2\phi \left( {\sigma_{bf} - \sigma_{sp} } \right)}}{{\sigma_{sp} + 2\sigma_{bf} + 2\phi \left( {\sigma_{bf} - \sigma_{sp} } \right)}}\sigma_{bf} $$

The Brownian motion of nanoparticles may be added to the Maxwell classical correlation in order to determine the effective and efficient heat conductivity of a nanofluid taking the account of the random movement of the nanoparticles in it as follows^58^:

16 $$ k_{nf} = k_{static} + k_{Brownian} $$

where,

$$ k_{static} = \frac{{k_{sp} + (n - 1)k_{bf} - (n - 1)(k_{bf} - k_{sp} )\phi }}{{k_{sp} + (n - 1)k_{bf} + (k_{bf} - k_{sp} )\phi }}k_{bf} ,k_{Brownian} = \frac{{\phi \rho_{p} \,c_{p,sp} }}{2}\sqrt {\frac{{2K_{B} \,T_{ref} }}{{3\,\pi \,d\,\mu_{nf} }}} $$

where *ψ* denotes sphericity, which is defined as the ratio of spherical surface area to real particle surface area with the same volumes, and *n* is the form factor of nanoparticles, expressed as *n* = 3/*ψ.* The reference temperature, *T*_*r*ef_; the Boltzmann constant, *K*_B_ = 1.38064852 × 10^−23^ JK^−1^; the blade, platelet, brick, cylinder, and spherical-shaped nanoparticles, and their diameters, *d*, are represented by the integers *n* = 8.6, 5.7, 4.9, 3.7, and 3.0, respectively.

The following transformation of variables (Eq. 7) is used to make the Eqs. (3)–(6) including boundary conditions (7)–(9) in the dimensionless form^57,58^:

17 $$ X = \frac{x}{L}\,,Y = \frac{y}{L},U = \frac{uL}{{\alpha_{bf} }},V = \frac{vL}{{\alpha_{bf} }},P = \frac{{pL^{2} }}{{\rho_{nf} \alpha_{bf}^{2} }},{\text{and}}\,\,\,\theta = \frac{{T - T_{c} }}{{T_{h} - T_{c} }} $$

The non-dimensionless governing equations by employing dimensionless variables are as follows^57,58^:

18 $$ \frac{\partial U}{{\partial X}} + \frac{\partial U}{{\partial Y}} = 0 \, $$

19 $$ U\frac{\partial U}{{\partial X}} + V\frac{\partial U}{{\partial Y}} = - \frac{{\rho_{bf} }}{{\rho_{nf} }}\frac{\partial P}{{\partial X}} + \Pr \left( {\frac{{\rho_{bf} }}{{\rho_{nf} }}} \right)\left( {\frac{{\partial^{2} U}}{{\partial X^{2} }} + \frac{{\partial^{2} U}}{{\partial Y^{2} }}} \right) $$

20 $$ U\frac{\partial V}{{\partial X}} + V\frac{\partial V}{{\partial Y}} = - \frac{{\rho_{bf} }}{{\rho_{nf} }}\frac{\partial P}{{\partial Y}} + \Pr \left( {\frac{{\rho_{bf} }}{{\rho_{nf} }}} \right)\left( {\frac{{\partial^{2} V}}{{\partial X^{2} }} + \frac{{\partial^{2} V}}{{\partial Y^{2} }}} \right) + \frac{{\left( {\rho \beta } \right)_{nf} }}{{\rho_{nf} \beta_{bf} }}{\text{Ra}}\Pr \theta - \frac{{\rho_{bf} }}{{\rho_{nf} }}\frac{{\sigma_{nf} }}{{\sigma_{bf} }}{\text{Ha}}^{2} \Pr \,V \, $$

21 $$ U\frac{\partial \theta }{{\partial X}} + V\frac{\partial \theta }{{\partial Y}} = \left( {\frac{{\alpha_{nf} }}{{\alpha_{bf} }}} \right)\left( {\frac{{\partial^{2} \theta }}{{\partial X^{2} }} + \frac{{\partial^{2} \theta }}{{\partial Y^{2} }}} \right) + \frac{{\alpha_{nf} }}{{\alpha_{bf} }}Q\theta $$

where, $\Pr = \frac{{\upsilon_{bf} }}{{\alpha_{bf} }}$ is the Prandtl number, ${\text{Ra}} = \frac{{g\beta_{bf} \left( {T_{h} - T_{c} } \right)L^{3} }}{{\upsilon_{bf} \,\alpha_{bf} }}$ is the Rayleigh number, ${\text{Ha}} = B_{o} L\sqrt {\sigma_{bf} /\mu_{bf} }$ is the Hartmann number, and $Q = \frac{{qL^{2} }}{{\alpha_{nf} \left( {\rho c_{p} } \right)_{nf} }}$ is the heat generation /absorption parameter.

Non-dimensional boundary conditions are as follows:

22 $$ {\text{At}}\;{\text{the}}\;{\text{horizontal}}\;{\text{wall}}:\quad U = 0,\,\,V = 0,\,\,\theta = \sin \,(\pi X) $$

22 $$ {\text{At}}\;{\text{the}}\;{\text{vertical}}\;{\text{wavy}}\;{\text{wall}}:\quad U = 0,\,\,V = 0,\,\,\theta = 0 $$

23 $$ {\text{At}}\;{\text{the}}\;{\text{inclined}}\;{\text{wall}}:\quad U = 0,\,\,V = 0,\,\,\frac{\partial \theta }{{\partial X}} = 0 $$

### Average Nusselt number

For this model, average Nusselt number on horizontal warmed wall has been calculated from the following expression^57,58^:

24 $$ {\text{Nu}}_{av} = - \left( {\frac{{k_{nf} }}{{k_{bf} }}} \right)\int\limits_{0}^{1} {\frac{\partial \theta }{{\partial Y}}} \,dX $$

## Computational procedure

The non-dimensional highly non-linear PDEs (18)–(21) including boundary conditions (22)–(24) are numerically solved using Galerkin weighted residual finite element technique that is a powerful tool to handle these kinds of non-linear equations. The information about the numerical technique is narrated by Zienkiewicz and Taylor^59^. In this numerical technique, the solution domain is discretized firstly within finite grid numbers that are composed of multiform triangle elements. Six node triangle shape elements are employed to improve the finite element equations. Then, the Galerkin weighted residual scheme is executed into a system of integral equations from non-linear PDEs. To solve these integral equations, Gauss’s quadrature method is also employed. Thereafter, the boundary necessary boundary conditions are employed to modify the non-linear algebraic. Newton–Raphson iteration method is employed for solving the global non-linear algebraic equation in terms of Matrix. The standard of convergence including error inference for the solution is worked as $\left| {\Upsilon^{m + 1} - \Upsilon^{m} } \right| \le 10^{ - 5}$, where $\Upsilon$ represents subsidiary variables $U,\,\,V,\,\,\theta$ and *m* represent iteration number.

### Grid independence test

**Figure 2:**
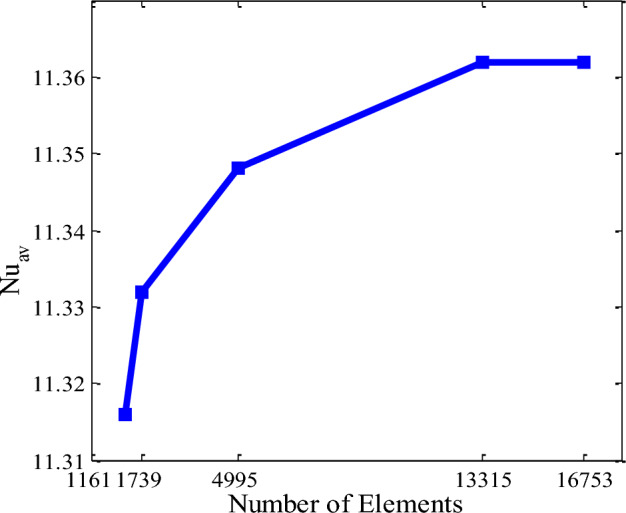
Average Nusselt number (Nu_av_) of different element numbers.

To guarantee the numerical scheme, a comprehensive mesh testing action is worked for the present problem of wavy triangular enclosure when Ra = 10^5^ with fixed Ha = 15, *ϕ* = 0.04, *Q* = 4, *λ* = 0, and *n* = 3. We observed the following five different grid systems designated as normal, fine, finer, extra fine, and extremely fine including elements number into the resolution field: 1161, 1739, 4995, 13,315, and 16,753. The numerical design is executed employing average Nusselt number (Nu_av_) for checking the grid fineness for the above-mentioned elements is displayed by Fig. 2. The elements 13,315 depicts less difference in the outcomes than the others elements for present study. Therefore, for the numerical simulation, elements 13,315 is founded for fulfil the requirements of the grid independent solution.

### Code validation

Through the data regarding average Nusselt number (Nu_av_), the current results are equated with Ghasemi et al.^60^ in order to verify the validity of the current numerical scheme using several Rayleigh numbers (Ra) and nanoparticle volume (*ϕ*) using Al_2_O_3_–H_2_O nanofluid with Ha = 30, Pr = 6.8377, and *n* = 3 (Table 2). In contrast, the same code was used for the present nanofluid model. It seems that using the current numerical system fulfills with requirements.

**Table 2:** Code authentication with Ghasemi et al.^60^ using of average Nusselt number when *n* = 3, Pr = 6.8377, and Ha = 30 for Al_2_O_3_–H_2_O nanofluid.

	Average Nusselt number (Nu_*av*_)								
	*ϕ* = 0			*ϕ* = 0.02			*ϕ* = 0.04		
	Ghasemi et al.^60^	Present study	Relative error (%)	Ghasemi et al.^60^	Present study	Relative error (%)	Ghasemi et al*.*^60^	Present study	Relative error (%)
10^3^	1.002	1.002	0	1.060	1.072	1.13	1.121	1.121	0
10^4^	1.183	1.182	0.08	1.212	1.235	1.90	1.249	1.269	1.60
10^5^	3.150	3.138	0.38	3.138	3.201	2.01	3.124	3.216	2.94
10^6^	7.907	7.815	1.16	7.979	8.018	0.49	8.042	8.087	0.56

## Results and discussion

This portion, we illustrate the outcomes of free convectional temperature transport and fluid flow of nanofluids into a wavy triangle shape enclosure by employing finite element method of Galerkin weighted residual type. The simulated outcomes form the numerical calculations of current problem is discussed for different model parameters designated as temperature generation/absorption parameter (*Q*), Rayleigh number (Ra), number of undulations (*λ*), volume fraction of nanoparticles (*ϕ*), Hartmann number (Ha), nanoparticles shape (*n*), size (*d*) and Brownian motion upon flow characteristics in isotherm contours, streamline patterns and average Nusselt number. The following domains of non-dimensional parameters for numerical computation are used: 10^4^ ≤ Ra ≤ 10^6^, 0 ≤ Ha ≤ 50, 0 ≤ *ϕ* ≤ 0.1, − 12 ≤ *Q* ≤ 12, 0 ≤ *λ* ≤ 3 and Pr = 6.8377, 1 nm ≤ *d* ≤ 100 nm, and *n* = 3, 3.7, 4.9, 5.7, and 8.6.

### Impact of Rayleigh number

**Figure 3:**
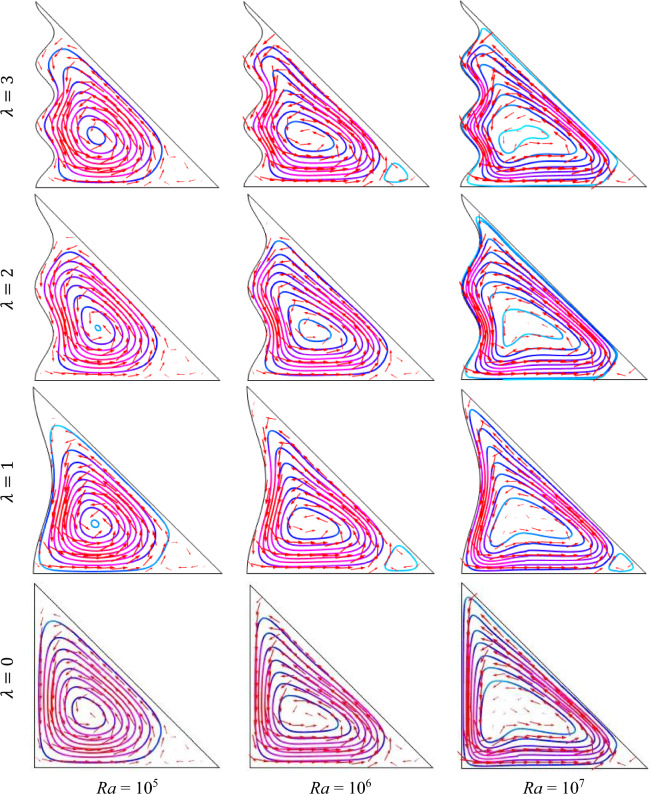
Outcomes of Rayleigh number (${\text{Ra}}$) for various undulation parameter (*λ*) using streamlines when *ϕ* = 0.04, Ha = 15, *n* = 3, *d* = 10 nm, and *Q* = 4.

The fluid's buoyancy forces are measured by the Rayleigh number. It measures how significant natural convection is in relation to other heat transfer mechanisms like forced convection and conduction. Figure 3 demonstrates outcomes of the Rayleigh number (Ra = 10^5^, 10^6^, 10^7^) using streamlines with fixed *ϕ* = 0.04, Ha = 15, *n* = 3, *d* = 10 nm and *Q* = 4. It is seen from this figure, for Ra = 10^5^, the streamline contours are not forcefully influenced by nanofluid particles while the sinusoidally warmed-up boundary is imposed on the horizontal wall which is a good syndrome of potential conduction. The form of streamline contours is nearly uniform for all undulation parameters (*λ*). As increases of buoyancy-driven parameter Ra (> 10^5^), fluid within the cavity is more debauched, and streamline contours capacity increases which indicates the convection mood of temperature transport. The streamlines also grab the maximum area within the enclosure for a higher Rayleigh number. In addition, for the increases of Ra, the denseness and vortex strength within the cavity are enhanced as well as turn familiar concerning the free convection. Also, for raising the value of the undulation parameter (*λ*) the extension of flow circulation turns out to impair with all Rayleigh numbers (Ra). This is due to the fact that the mounting value of *λ* produces a friction force between the wavy surface and the liquid that generates temperature which diminishes liquid rapidity within the enclosure.

**Figure 4:**
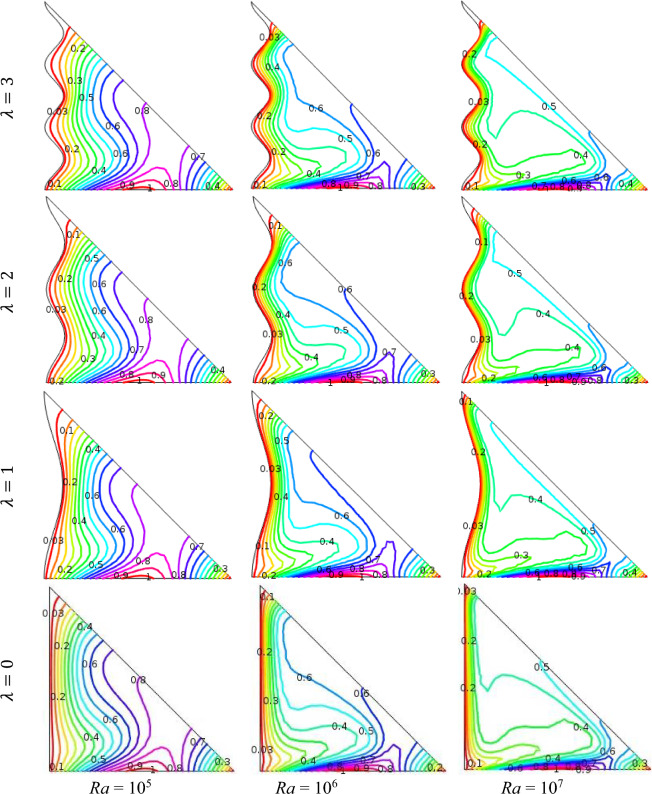
Outcomes of Rayleigh number (Ra) for various undulation parameter (*λ*) using isotherms when *ϕ* = 0.04, Ha = 15, *n* = 3, *d* = 10 nm, and *Q* = 4.

Isotherms are useful to visualize the temperature flow style whether it is convection or conduction. It is also extremely efficient to measure the proficiency of temperature transference into nanofluid. Figure 4 depicts Rayleigh number influence (Ra = 10^5^, 10^6^, 10^7^) on isotherms with fixed *ϕ* = 0.04, Ha = 15, *n* = 3, *d* = 10 nm and *Q* = 4. These figures illustrate that for low Ra, streamlines are superfluous compact near the bottom hot and perpendicular wavy cold walls into the enclosure for conduction type of temperature disposal. It is also seen that for the Rayleigh number, the isotherm's contours are parabolic in shape near horizontal hot walls on account of conduction.

The pattern of the isotherm contours as well as heated field become non-persistent with the variation of Ra for a fixed undulation parameter (*λ*). As the buoyancy driven parameter augments Ra (> 10^5^), the crowded of isotherm contours into the cavity enhances and intimates neighbor horizontal hot and wavy cold walls which indicates that the formation of heated boundary layer on horizontal wall for conduction style of temperature changing. It is also seen that a dilute heated boundary is elaborated near perpendicular wavy wall. The wavy wall of the triangular domain can improved by the turbulence and vortices that accelerates the heat transfer rate in the nanofluid. It is also seen that better thermal performance is observed from the wavy surface's acceleration of nanofluid mixing as compared to a triangular cavity with plain walls. Because of the higher flow resistance caused by wavy walls, the pressure drop in the flow is also enhanced. Furthermore, isotherm contours are more compact and concentrate when undulation number increases. The physical significance is that a high Rayleigh number implies that buoyancy-driven convection dominates.

### Impact of Hartmann number

**Figure 5:**
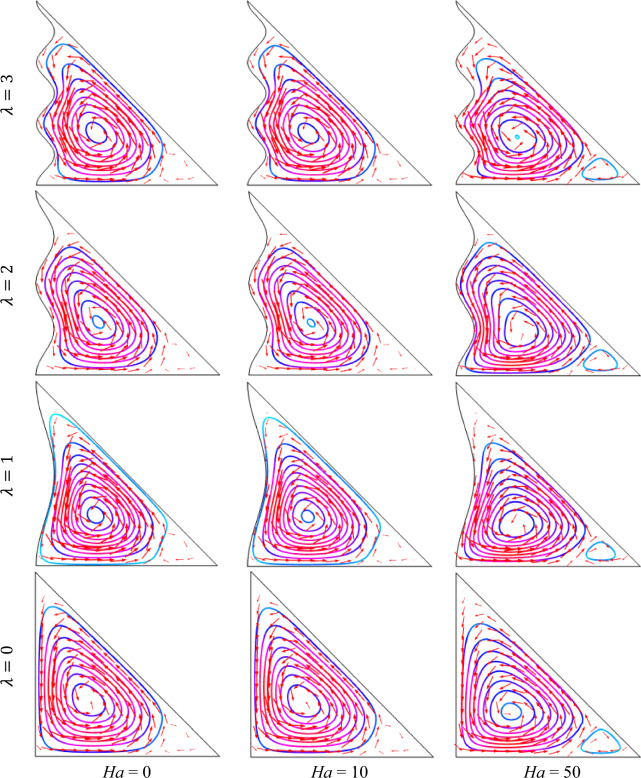
Outcomes of Hartmann number (Ha) for various undulation parameter (*λ*) using streamlines when *ϕ* = 0.04, Ra = 10^5^, *n* = 3, *d* = 10 nm, and *Q* = 4.

The outcomes of Hartmann number Ha (= 0, 10, 50) on streamlines with fixed *ϕ* = 0.04, Ra = 10^5^, *n* = 3, *d* = 10 nm, and *Q* = 4 (Fig. 5). It is seen from the figure for the absence of the Hartmann number (Ha = 0) a clockwise circulation is formed nearly the whole enclosure. A tiny revolving chamber with a vortex is also generated at the center of the enclosure. As increases Ha, the pattern of the streamline has been changed and flow strength diminishes within the cavity. Because an external strong magnetic field generates a Lorentz force that weakens the streams within the enclosure. This figure also depicts that a small secondary counterclockwise vortex is created on the right corner of the enclosure for a higher Hartmann number. This is due to the fact that the flow enlarges in the middle and this leads to create a secondary tiny circulation cell inside the enclosure. These results also narrate that adjacent fluid near the bottom hot wall after getting more heated, and attempts to go overhead by virtue of buoyant force whilst comparatively refrigerant liquid neighbor cooled wavy wall moves to the horizontal hot wall which creates a secondary small vortex. In addition, for the increasing values of undulation parameter λ (= 1, 2, 3), a tiny circulation grows neighbor to the horizontal hot wall. As increases *λ*, the enclosure kernel fills with more streamlines. It is also seen that the alternation in *λ* causes an ordinary change in streamlines for a particular Hartmann number.

**Figure 6:**
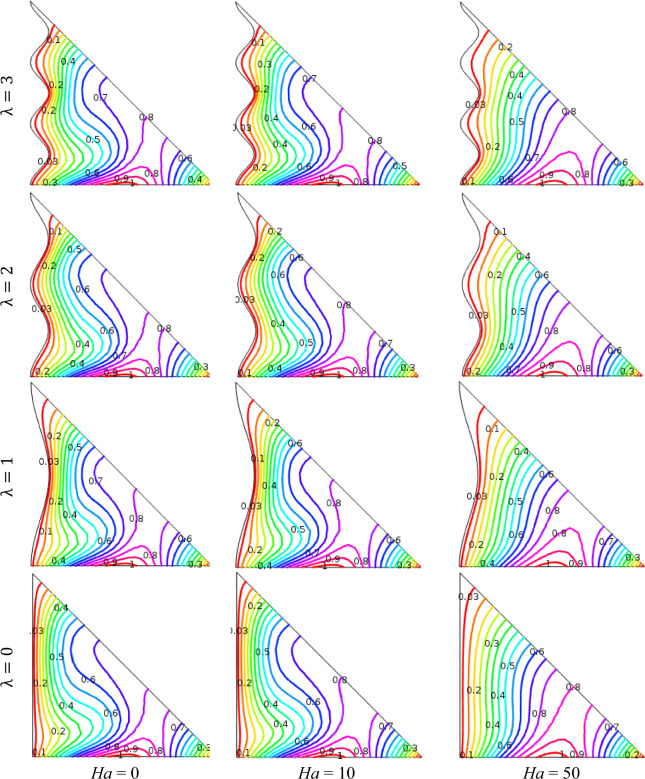
Outcomes of Hartmann number (Ha) for various undulation parameter (*λ*) using isotherms when *ϕ* = 0.04, Ra = 10^5^, *n* = 3, *d* = 10 nm, and *Q* = 4.

The impact of Ha (= 0, 10, 50) using distributions of isotherms is demonstrated in Fig. 6 with fixed *ϕ* = 0.04, Ra = 10^5^, *n* = 3, *d* = 10 nm, and *Q* = 4. The figures depict that isotherm lines are compact neighbor horizontal hot wall and vertical wavy cold wall inside the enclosure because Ha creates interior incident into the fluid that generates more temperature into the fluid. In the absence of an external magnetic effect (Ha = 0), isotherm contour patterns are more conspicuous which indicates that strong convection occurs for the nonappearance of the magnetic field. For higher Hartmann numbers, isotherm contour lines walk towards the vertical wavy surface and are almost parallel to each other. This is due to the fact that the strong Lorentz force from the Hartmann number reduces the temperature distribution. Besides, for a particular Hartmann number, a higher temperature transport is observed at *λ* = 3 than other values of the undulation parameter (*λ*).

Therefore, for upper values of *λ* and lower Hartmann numbers, the isothermal contours are slightly denser near the wavy surface. A magnetic field's strength is an important factor that have varying effects on heat transfer rates, flow patterns, nanoparticle behavior, and energy efficiency in the context of nanofluid in a wavy triangular cavity. The velocity of charged nanoparticles within the nanofluid can be affected by a magnetic field. The convective motion is often suppressed by the Lorentz force acting on the charged nanoparticles. Because nanofluids have higher thermal conductivity when subjected to magnetic fields, these properties can change heat transfer in nanofluids. The alignment and motion of nanoparticles are influenced by the magnetic field, which optimizes heat transfer rates. The dispersion of nanoparticles in the nanofluid can also be impacted by the magnetic field. By preventing or controlling agglomeration, it can assist keep nanoparticles evenly distributed throughout the fluid. This is essential to preserving the nanofluid's essential characteristics.

### Impact of heat generation/absorption

**Figure 7:**
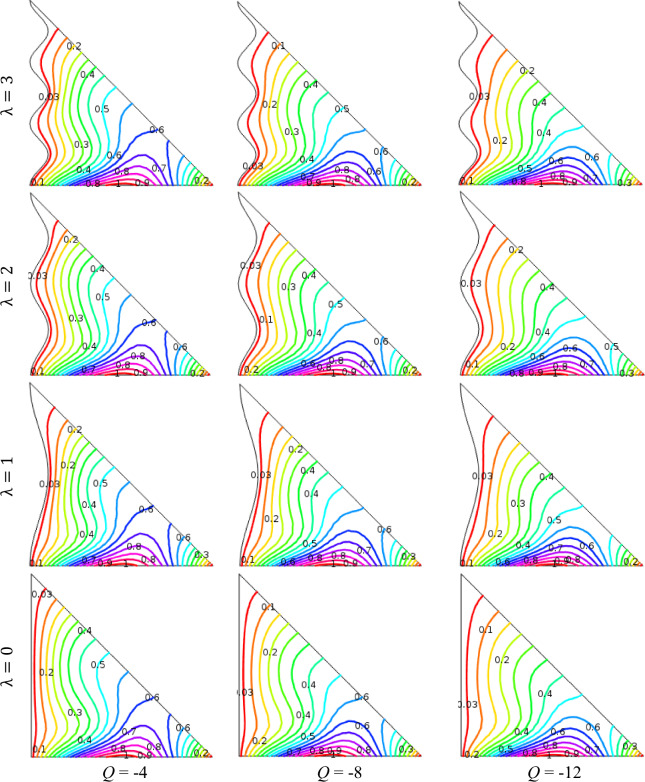
Outcomes of *Q* (< 0) for various undulation parameter (*λ*) using isotherms when *ϕ* = 0.04, Ha = 15, Ra = 10^5^, *n* = 3, and *d* = 10 nm.

**Figure 8:**
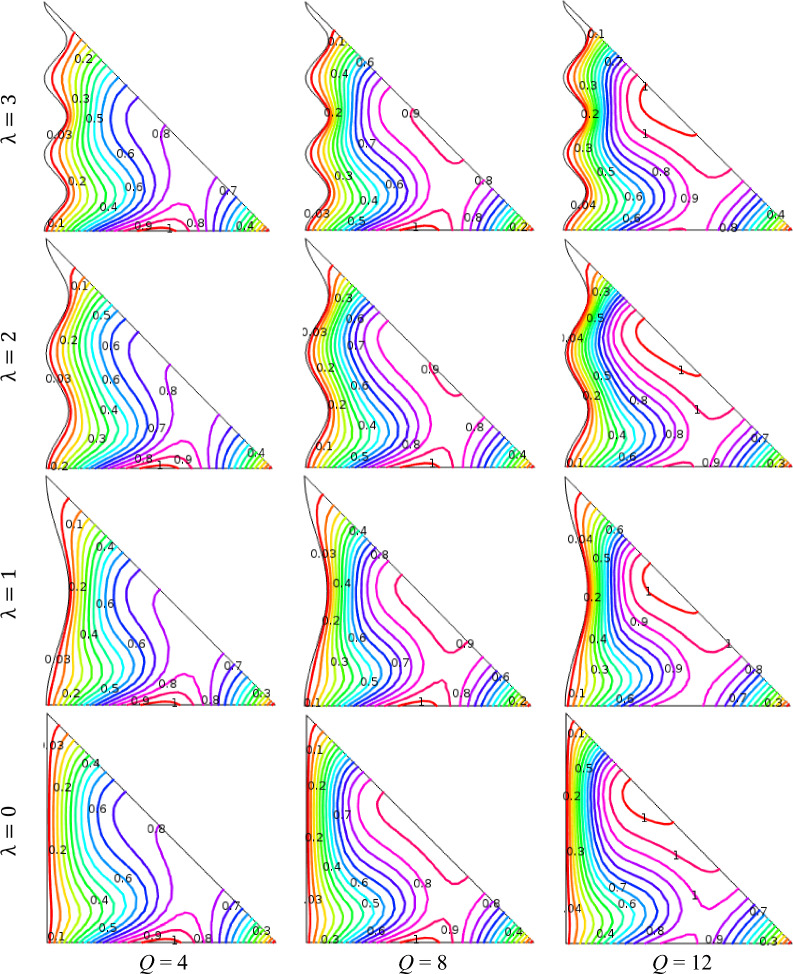
Outcomes of *Q* (> 0) for various undulation parameter (*λ*) using isotherms when *ϕ* = 0.04, Ha = 15, Ra = 10^5^, *n* = 3, and *d* = 10 nm.

The effects of heat generation *Q* (> 0) or heat absorption *Q* (< 0) on isotherm contours for variation of wave number *λ* (= 0, 1, 2, 3) when *ϕ* = 0.04, Ha = 15, Ra = 10^5^, *n* = 3, and *d* = 10 nm are presented in Figs. 7 and 8, respectively. The compactness of the isotherms is denser near the hot and cold wall for the heat sink than to the heat source parameter. We observed that the denseness of the isotherms within the enclosure for the heat sink condition *Q* = − 12. But, the crowded isotherm lines are diminished slowly for non-dimensional temperature enhanced up to the higher values of heat generation case *Q* = 12. We also observed that for temperature absorption situations, enclosure heat is lesser compared to the temperature in fluid including heat generation. The temperature gradient near the enclosure wavy wall with the heat source case is inferior compared to the heat sink case. This phenomenon is observed for all undulation parameters (*λ*). The heat source increases fluid temperature and reduces temperature differences inside the enclosure. In addition, heat sink enhances, isotherm contours compact neighbor the wavy surface within the cavity. Also, isotherm contours are parabolic in appearance neighboring the warmed-up wall and a dilute heated boundary layer is developed neighbor the wall within the cavity.

### Effect of average Nusselt number

**Figure 9:**
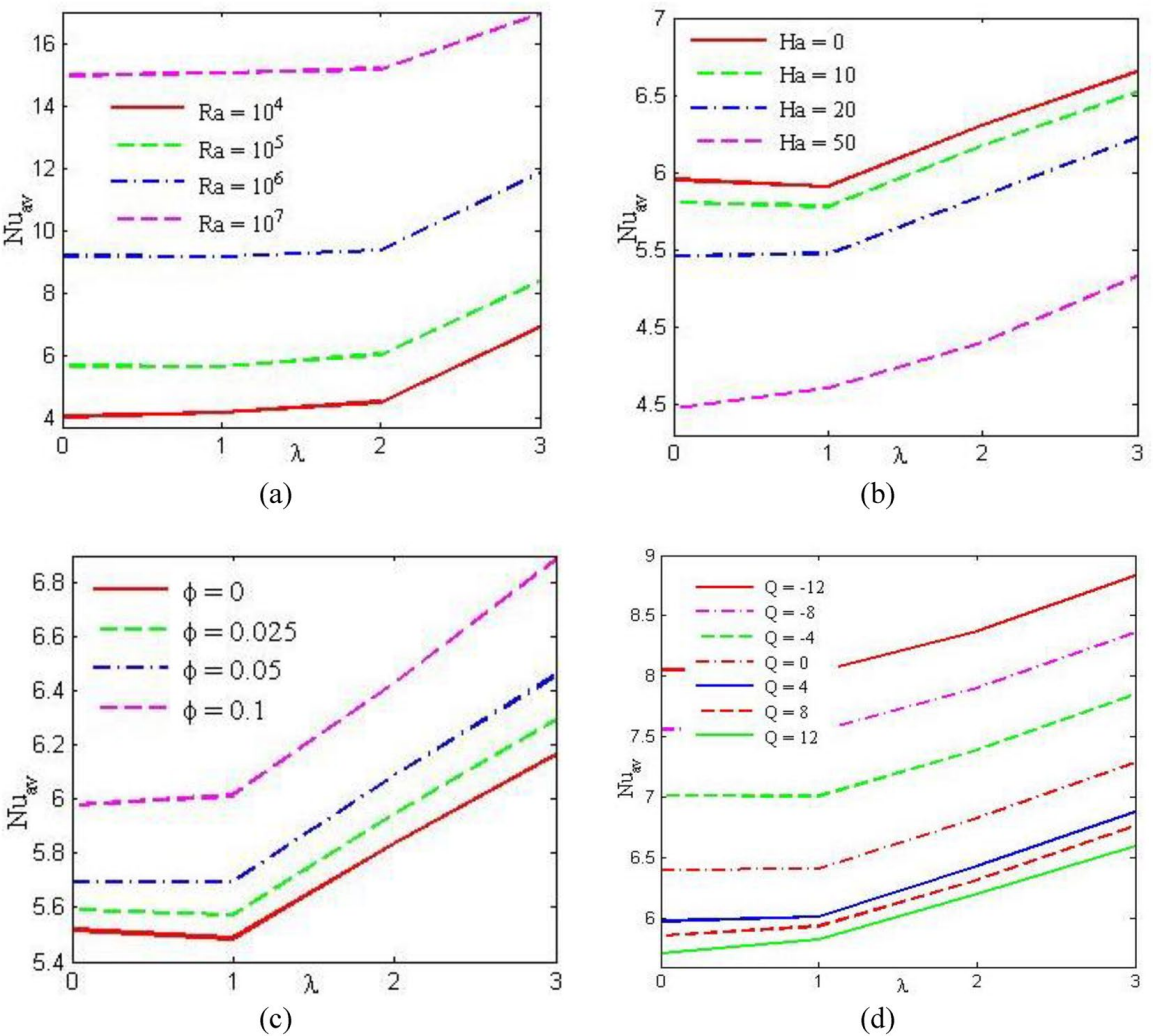
Average Nusselt number for (**a**) Rayleigh number, (**b**) Hartman number, (**c**) nanoparticles volume fraction and (**d**) Heat generation or absorption parameter.

Figure 9, respectively, demonstrates the influence of mean Nusselt number (Nu_av_) along the bottom hot wall varying (a) Rayleigh number (Ra), (b) Hartmann number (Ha), (c) Nanoparticles volume (*ϕ*) and (d) heat generation/absorption (*Q*) against the wave number, *λ* (= 1, 2, 3) taking as default values other parameters. Figure 9a, depicts that the average rate of temperature transfer increases for all values of buoyancy-driven parameter Ra. It is also seen that the temperature transfer rate is enhanced effectively with the augmentation of wave number *λ*. Therefore, the average rate of temperature transfer is more efficient for ascending values of the undulation parameter (*λ*). In addition, the enhancement of Ra promotes the buoyancy effect, therefore a large quantity of temperature is shifted into the enclosure from the horizontal warmed wall. Furthermore, the average rate of temperature transfer increases gradually by virtue of the corrugated surface of the enclosure. To get the required heat production or absorption properties, one can adjust the nanofluid design, nanoparticle type, concentration, and size. In order to maximize heat transfer and energy efficiency in a variety of industries, including chemical processes, microelectronics cooling, the oil and gas sector, solar thermal collectors, heat exchangers, and cooling systems, researchers are still investigating and creating novel nanofluids with particular qualities.

Figure 9b depicts the mean Nusselt number (Nu_av_) reduced for the augmentation of Ha. For Ha, Nu_av_ constantly turns higher within the enclosure. The Hartmann number (Ha) produces a strong Lorentz force which acts in opposition to the velocity fields and reduces the circulation strength into fluid within the cavity. Concerning the ascending as well as the collapsing model for wavy surface, the outcomes represent Nu_av_ increases sufficiently for the improvement of the undulation parameter (*λ*). Figure 9c depicts that as nanoparticle volume (*ϕ*) rises, Nu_av_ also rises monotonically. Therefore, the composition of nanoparticles in base fluid enhances Nu_av_. Figure 9d depicts heat generation or heat absorption rises for ascending values of *λ*. This figure also depicts that higher Nu_av_ is observed for the heat sink. The temperature transport rate reduces for the rising heat source. In contrast, the temperature transfer rate is enhanced with the increase of heat sink. In total, the undulated surface enhances the mean Nusselt number (Nu_av_). In comparison to a cavity with a flat wall, a triangular cavity with a wavy wall can improve heat transmission, but it also creates issues with pressure drop, nanofluid stability, and flow pattern development. For nanofluid flow and heat transfer, a triangular hollow with a wavy or corrugated wall adds complexity and can significantly affect the flow and heat transfer properties. Heat transfer may be improved by the turbulence and vortices that the wavy wall geometry may produce in the nanofluid flow. Better thermal performance results from the wavy surface's acceleration of nanofluid mixing as compared to a triangular cavity with plain walls. Furthermore, because of the higher flow resistance caused by wavy walls, the pressure drop in the flow is also enhanced. The flow velocity and the particular size and waviness of the walls determine the effect on pressure drop. Moreover, the stability of the nanofluid may be impacted by the triangular enclosure with a wavy wall. Because of their propensity to aggregate or settle out of suspension, nanoparticles can affect the flow phenomena caused by irregular flow patterns. The flow domain's boundary layer growth can also be affected by the wavy wall geometry.

### Effect of nanoparticles shape, size and Brownian motion

In order to investigate how Brownian motion influences the rate of heat transfer, the heat transport rate in regard to the average Nusselt number along the heated diameter is computed. Table 3 examines the effects of the Brownian effect, volume (ϕ), shape (n), and size (d) of the nanoparticles, with fixed values of λ = 3, Ha = 15, and Ra = 10^5^. The result suggests that Brownian motion is a significant factor in the enhancement of the temperature transfer rate. The Brownian motion of the nanoparticles adds to the transmission of additional heat in the nanofluids and to the micro-convection of the fluid surrounding individual nanoparticles as a result of their movement into the base fluid. The outcome demonstrates that, for both scenarios without/with the Brownian motion of the nanoparticles, the effect of the volume fraction of the nanoparticles on the average Nusselt number is more effective for considering the Brownian effect. Furthermore, when taking into account the Brownian motion of the nanoparticles, the average heat transfer increases by 86.77% for 5% nanoparticle volume fractions while without the Brownian effect, it increases by only 5.52%.

**Table 3:** Variation of average Nusselt number (Nu_*av*_) along heated wall for different nanoparticles volume (*ϕ*), diameter (*d*) and shape (*n*) of nanoparticles considering with/without Brownian with Ra = 10^5^, Ha = 15, and λ = 3.

Brownian effect (BM)	Nanoparticles volume (*ϕ*)	Nanoparticles shape (*n*)	Nanoparticles diameter (*d*)	Average Nusselt number (Nu_av_)	Increases (%)
with BM	0	3.0	10 nm	4.267974931830414	–
	0.01			5.140180145823340	20.43
	0.02			5.930500451043041	38.95
	0.05			7.971343224154655	86.77
	0.04	3.0	10 nm	7.254760603941890	–
		3.7		7.334404774458736	1.10
		4.9		7.470160718151543	2.97
		5.7		7.560131307489343	4.21
		8.6		7.882830676803941	8.66
	0.04	3.0	100 nm	5.398074838809371	–
			50 nm	5.779106109891497	7.06
			20 nm	6.519641772718621	20.78
			10 nm	7.334404744750127	35.87
			1 nm	13.02908943936327	141.37
Without BM	0	3.0	–	4.267974931830413	–
	0.01		–	4.309365483763966	0.97
	0.02		–	4.353923846604179	2.01
	0.05		–	4.503711767742641	5.52
	0.04	3.0	–	4.362500816778986	–
		3.7	–	4.451326343833879	2.03
		4.9	–	4.602177643914426	5.49
		5.7	–	4.701783415723639	7.78
		8.6	–	5.056793437004171	15.92

This Table also shows that the average rate of heat transfer decreases with the increase of the diameter of nanoparticles. The small size of nanoparticles is more effective for the enhancement of thermal performance. The physical meaning of it, by decreasing the nanoparticle diameter, the specific area increases, which helps to enhance nanofluid thermal conductivity and consequently increases the average Nusselt number. The heat transport rate increases by 20.78% when the nanoparticle diameter reduces 100 nm to 20 nm, it increases by 35.87% when the nanoparticle size reduces 100 nm to 10 nm, and it rises by 141.37% when the nanoparticle diameter reduces 100 nm to 1 nm. The blade shape of nanoparticles generates the highest thermal performance. The heat transport rate increases by 8.66% for the blade shape of nanoparticles compared to the spherical shape of nanoparticles. In many different industries, including heat exchanger design, solar energy systems, chemical engineering, renewable energy systems, microfluidics, and so on, the study of heat generation/absorption effects on natural convective heat transfer in wavy triangular cavities filled with nanofluids has a wide range of practical applications. This research helps to improve energy efficiency, system performance, and the development of novel technologies.

## Conclusions

The two-dimensional time independent natural convection laminar flow and temperature transport in a sinusoidally wavy triangle loaded by cupper-water nanofluid in the presence of heat generation/absorption parameter is investigated numerically. Galerkin weighted residual finite element technique is executed for numerical calculations. The impacts of several model parameters such as heat generation/absorption parameter (*Q*), Rayleigh number (Ra), number of undulations (*λ*), volume fraction of nanoparticles (*ϕ*), Hartmann number (Ha), nanoparticles shape (*n*), size (*d*) and Brownian motion on the flow and thermal field are presented using isotherm lines, streamline contours and average Nusselt number and interpreted them. The following major findings are listed:

- The wavy surface has a significant impact on the flow domain as well as the temperature field. The ascending number of wave surfaces (*λ* = 3) augments temperature transfer sufficiently than the flat surface (*λ* = 0).
- The strength of isotherms reduces successively for the rise of heat generation up to high heat generation (*Q* = 12). Consequently, higher temperature into nanofluid within the enclosure that reduces heat transfer rate.
- By the increases of Rayleigh number and nanoparticles volume, fluid flow as well as temperature flow change remarkably. The higher Rayleigh number and lower Hartmann number correspond to convection heat transfer.
- The small size of nanoparticles has a key role in thermal transport. The thermal transport rate increases by 35.87% when the nanoparticle diameter reduces from 100 to 10 nm.
- A major contributing component to the improvement of heat transmission is Brownian motion. The heat transport rate increases by 20.43% for considering the Brownian effect for only 1% of nanoparticle volume.
- The blade shape of nanoparticles confirms better thermal performance. The thermal transport increases by 8.66% compared to the default spherical shape of nanoparticles.

## Data Availability

The datasets used and analyzed during the current study are available from the corresponding author on reasonable request.

## List of symbols

A: Wave amplitude [m]
*B*_0_: Magnitude of magnetic field [kgs^−^^2^A^−^^1^]
*c*_*p*_: Specific heat at constant pressure [Jkg^−^^1^K^−^^1^]
*g*: Gravitational acceleration [ms^−^^2^]
Ha: Hartmann number
*k*: Thermal conductivity [Wm^−^^1^K^−^^1^]
*L*: Length of the enclosure at bottom wall
Nu_*av*_: Average Nusselt number
*p*: Dimensional pressure [kgm^−^^1^s^−^^2^]
*P*: Dimensionless pressure
Pr: Prandtl number
Ra: Rayleigh number
*T*: Fluid temperature [K]
*u, v*: Dimensional velocity components [ms^−^^1^]
*U, V*: Dimensionless velocity components
*x, y*: Dimensional coordinates [m]
*X, Y*: Dimensionless coordinates

## Greek letters

*α*: Thermal diffusivity [m^2^s^−^^1^]
*β*: Thermal expansion coefficient [K^−^^1^]
*ϕ*: Volume fraction of nanoparticles
*μ*: Dynamic viscosity [kgm^−^^1^s^−^^1^]
*υ*: Kinematic viscosity [m^2^s^−^^1^]
*θ*: Non-dimensional temperature
*ρ*: Density [kgm^−^^3^]
*σ*: Electric conductivity
*λ*: Undulation number

## Subscripts

*h*: Hot surface
*c*: Cold surface
*nf*: Nanofluid
*sp*: Solid particle
*bf*: Base fluid
