# Author Correction: Heat generation/absorption effect on natural convective heat transfer in a wavy triangular cavity filled with nanofluid

**DOI:** 10.1038/s41598-024-67493-w

**Published:** 2024-07-19

**Authors:** Tarikul Islam, Md. Nur Alam, Shafullah Niazai, Ilyas Khan, Md. Fayz-Al-Asad, Sultan Alqahtani

**Affiliations:** 1 CMUP, University of Porto, Porto, Portugal; 2 Department of Mathematics, Bangabandhu Sheikh Mujibur Rahman Science and Technology University, Gopalganj, 8100 Bangladesh; 3 Department of Mathematics, Pabna University of Science and Technology, Pabna, 6600 Bangladesh; 4 Department of Mathematics, Education Faculty, Laghman University, Mehtarlam City, Laghman 2701 Afghanistan; 5 Department of Mathematics, College of Education, Majmmah University, 11952 Al-Majmaah, Saudi Arabia; 6 Department of Mathematics, American International University-Bangladesh, Kratoli, Khilkhet, Dhaka, 1229 Bangladesh; 7 Department of Mathematics, Bangladesh University of Science and Technology (BUET), Dhaka, 1000 Bangladesh; 8 College of Engineering Mechanical Engineering Department, King Khalid University, Abha, Saudi Arabia

Correction to: *Scientific Reports* 10.1038/s41598-023-48704-2, published online 01 December 2023

The original version of this Article contained errors in the Affiliations.

Md. Nur Alam was incorrectly affiliated with ‘Department of Mathematics, Bangabandhu Sheikh Mujibur Rahman Science and Technology University, Gopalganj, 8100, Bangladesh.’

The correct affiliation is listed below:

Department of Mathematics, Pabna University of Science and Technology, Pabna-6600, Bangladesh.

Additionally an affiliation for Tarikul Islam was omitted. The correct affiliations are listed below:

CMUP, University of Porto, Porto, Portugal

Department of Mathematics, Bangabandhu Sheikh Mujibur Rahman Science and Technology University, Gopalganj, 8100, Bangladesh

Furthermore, an affiliation for Md. Fayz-Al-Asad was omitted. The correct affiliations are listed below:

Department of Mathematics, American International University-Bangladesh, Kratoli, Khilkhet, Dhaka-1229, Bangladesh.

Department of Mathematics, Bangladesh University of science and technology (BUET), Dhaka-1000, Bangladesh.

The original Article has been corrected.

